# Functional study on the mutations in the silkworm (*Bombyx mori*) acetylcholinesterase type 1 gene (*ace*1) and its recombinant proteins

**DOI:** 10.1007/s11033-013-2877-8

**Published:** 2013-12-10

**Authors:** Ju-mei Wang, Bin-bin Wang, Yi Xie, Shan-shan Sun, Zhi-ya Gu, Lie Ma, Fan-chi Li, Yi-fan Zhao, Bin Yang, Wei-de Shen, Bing Li

**Affiliations:** 1 School of Basic Medicine and Biological Sciences, Soochow University, Suzhou, 215123 Jiangsu People’s Republic of China; 2 National Engineering Laboratory for Modern Silk, Soochow University, Suzhou, 215123 Jiangsu People’s Republic of China; 3 Jiangsu Minxing Cocoon Silk Stock Co., Ltd, Dongtai, 224200 Jiangsu People’s Republic of China

**Keywords:** *Bombyx mori*, Acetylcholinesterase, Gene mutation

## Abstract

The acetylcholinesterase of Lepidoptera insects is encoded by two genes, *ace*1 and *ace*2. The expression of the *ace*1 gene is significantly higher than that of the *ace*2 gene, and mutations in *ace*1 are one of the major reasons for pesticide resistance in insects. In order to investigate the effects of the mutations in *ace*1’s characteristic sites on pesticide resistance, we generated mutations for three amino acids using site-directed mutagenesis, which were Ala(GCG)303Ser(TCG), Gly(GGA)329Ala(GCA) and Leu (TCT)554Ser(TTC). The Baculovirus expression system was used for the eukaryotic expression of the wild type *ace*1 (*wace*1) and the mutant *ace*1 (*mace*1). SDS-PAGE and Western blotting were used to detect the targeting proteins with expected sizeof about 76 kDa. The expression products were purified for the determination of AChE activity and the inhibitory effects of physostigmine and phoxim. We observed no significant differences in the overall activity of the wild type and mutant AChEs. However, with 10 min of physostigmine (10 μM) inhibition, the remaining activity of the wild type AChE was significantly lower than that of the mutant AChE. Ten min inhibition with 33.4 μM phoxim also resulted in significantly lower remaining activity of the wild type AChE than that of the mutant AChE. These results indicated that mutations for the three amino acids reduced the sensitivity of AChE to physostigmine and phoxim, which laid the foundation for future in vivo studies on AChE’s roles in pesticide resistance.

## Introduction

Acetylcholinesterase (AChE, EC 3.1.1.7) is encoded by the acetylcholinesterase gene (*ace*), and it maintains the normal transmission of neural impulses in synaptic clefts through catalyzing the hydrolysis of neurotransmitter acetylcholine. AChE is the target enzyme for organophosphorus and carbamate pesticides [1]. These pesticides can bind to AChE and reduce its activity, leading to massive accumulation of postsynaptic membrane acetylcholine, continuous stimulation, biological convulsion and the eventual insect death.

The *ace* gene’s overexpression and mutations are the main reason of pesticide resistance to organophosphate pesticides. Gao and Zhu found that increased transcription rates and/or stability of the mRNA resulted in elevated levels of AChE mRNA in OP-resistant clones of the greenbug (*Schizaphis graminum*) [2]; the pesticide resistance was increased by the loss of 3 or 5 glutamine residues on the C-terminus of AChE that increased GPI anchor efficiency to recruit more AChE on the plasma membrane. Increased number of GPI-anchored molecules in synaptic clefts may reduce their sensitivity to insecticides [3]. However, it is more widely believed that point mutations accompanied with the changes in kinetic parameters are the major reason of pesticide resistance [4–7]. Such resistance-related point mutations include the substitutions of key sites in the activity region that have spatial effects, which may change the directivity of active site residues. Indeed, many potential point mutations in the *ace* gene have been shown to result in the resistance to organophosphorus pesticides [8, 9].

Cao et al. [10] found that the expression level of *ace*2 was actually higher than that of *ace*1 in *B. mori* cell lines. The *ace*1 gene plays an important role in the cholinergic function and serves as the main target of anticholinesterase insecticides in insects [11]. Baek et al. [12] has reported that the organophosphate-resistant strain of Lepidoptera diamondback moth (*Plutella xylostella*) *ace*1 possessed mutations for three amino acids (Ala201Ser, Gly227Ala, and Ala441Gly) in its conserved sequence, which may confer the moths their pesticide resistance. Amino acid sequence analysis revealed that the mutation of Ala441Gly also exists in the *ace*1 gene in *B. germanica*; however, Kim et al. [13] reported that *B. germanica* was not resistant to organophosphorus pesticides. Therefore, this mutation may only play very limited role, if any, in pesticide resistance. Li et al. [14] discovered that the Leu452Ser mutation in the *ace*1 gene is unique in the resistant strains of *P. xylostella* (ACCESSION: AY970293) by comparing the sequences of different insect *ace*1s, suggesting important correlation between this site and insecticide resistance.

The silkworm (*Bombyx mori*) is an important economic insect and has also become an important model insect with its finished genomic maps and re-sequencing [15–17]. In 1999, Surendra Nath and Surendra Kumar [18] first used *B. mori* as a model to study the effects of organophosphate insecticides on AChE’s activity and metabolic levels. In 2007, Seino et al. [19] first cloned and characterized the full length cDNA of the two *ace* genes in *B. mori*. Shang et al. [20] first expressed the *B. mori* Bm-*ace*1 and Bm-*ace*2 in eukaryotic cells and demonstrated that the eukaryotic Bm-*ace*2 was sensitive to organophosphorus pesticides. In 2009, Chen et al. [21] reported the activity of *B. mori* AChE and the transcript differences of *ace*1 and *ace*2. Peng et al. [22] revealed the transcript level changes of *ace* genes induced by different doses of pesticides, indicating *ace*1’s important role in pesticide induction. However, no studies have been reported on the functional roles of the mutant sites in *B. mori* *ace*1 on organophosphorus pesticide resistance.

## Materials and methods

### Materials and major reagents

*Escherichia coli* DH5α and Top10 strains were maintained in our laboratory. The Bac to Bac expression system, Bacmid-containing *E. coli* strain Ac DH10Bac and the pFastBac™ HT B plasmid were kindly provided by Professor Wenbing Wang at Jiangsu University. The *sf*9 cells were subcultured in our laboratory.

The DNA polymerase, restriction endonucleases, T4 DNA ligase, and DNA marker were purchased from TaKaRa Biotechnology (Dalian) Co., Ltd. The *Pfu* DNA polymerase and protein prestained marker were purchased from Fermentas. PCR primers were synthesized in Shanghai Sangon Biological Engineering Technology & Services Co., Ltd. The primary anti-His-tag antibody rabbit IgG and the secondary AP-labeled goat anti-rabbit IgG were purchased from Abcam Co., Ltd., UK and Santa Cruz Biotech, Inc, USA, respectively. AP Conjugate Substrate Kit (170-6432) was purchased from Bio-Rad Laboratories. Insect cell culture medium TC-100 was purchased from Applichem. Fetal bovine serum (FBS) was purchased from Hyclone; and Celfectin transfection reagent and the Ni–NTA purification system were purchased from Invitrogen. The chemical Phoxim [O,O-Diethyl O-(alpha-cyanobenzylideneamino)phosphorothioate] was purchased from Sigma-Aldrich Company. The physostigmine was purchased from Tokyo Kasei Kogyo Co., Ltd. Other major reagents were purchased from Shanghai Sangon Biological Engineering Technology & Services Co., Ltd.

### Methods

#### *Ace*1 cloning and site-directed mutagenesis

Three pairs of mutagenesis primers were designed (Table 1) to amplify the PCR product containing Ala(GCG)303Ser(TCG), Gly(GGA)329Ala(GCA) and Leu (TCT)554Ser(TTC) mutation. The Ala303Ser mutation was gernerated by using pUC-*wace*1 plasmid as template, F1 and R1 as primers, and *Pfu* high fidelity enzyme as DNA polymerase. The PCR product was digested with *Dpn*I to eliminate the methylated DNA template but retain the mutant PCR product. Then the product was cloned into pUCmT vector and confirmed by sequencing. F2, R2; F3, R3 primers were used to further amplify the mutant fragment and confirmed by sequencing.

**Table 1:** Primer sequences of PCR

The name of primer	Primer sequence	Length of product (bp)
F1	TTATTCGGTGAATCATCGGGAGCCGTGTCAG	4,800
R1	CTGACACGGCTCCCGATGATTCACCGAATAA	
F2	CTATCATGCAGTCTGCAGCCGCCACTGCTCC	4,800
R2	GGAGCAGTGGCGGCTGCAGACTGCATGATAG	
F3	GTTCGGGGAGCCTTCCAATCCCGGGAAA	4,800
R3	TTTCCCGGGATTGGAAGGCTCCCCGAAC	
*ace*1-*Xba* I	GCTCTAGAATGCGCGTGGTGTTGGCA	2,100
*ace*1-*Xho* I	CCGCTCGAGTTATATGGTGTATTTGAACAGTGC	
M13 forward	GTTTTCCCAGTCACGAC	4,530
M13 reverse	CAGGAAACAGCTATGAC	

#### Construction and identification of recombinant vector pFastBac™ HT B-*ace*1

The *ace*1-*Xba*I and *ace*1-*Xho*I primers containing restriction endonuclease sites (Table 1) were designed to amplify the *ace*1 fragment from pUC-*wace*1 and pUC-*mace*1, which was later digested by *Xba*I and *Xho*I and ligated into pFastBac™ HT B. After transformation into the *E. coli* DH5α competent cells, positive recombinant plasmids pFastBac™ HT B-*wace*1 and pFastBac™ HT B-*mace*1 were selected and digested with *Xba*I and *Xho*I to confirm the correct insertion of pFastBac™ HT B.

#### Construction and identification of recombinant baculovirus plasmids Bacmid-*wace*1 and Bacmid-*mace*1

Recombinant plasmids pFastBac™ HT B-*wace*1 and pFastBac™ HT B-*mace*1 were extracted and transformed into *E. coli* Ac DH10Bac competent cells. Recombinant bacmids Bacmid-*wace*1 and Bacmid-*mace*1 were extracted by the alkali lysis method with blue colonies as negative controls. The upstream and downstream M13 primers were used to identify and screen for the targeting recombinant plasmids Bacmid-*wace*1 and Bacmid-*mace*1.

#### Transfection of recombinant baculovirus plasmids Bacmid-*wace*1 and Bacmid-*mace1* into *sf*9 cells

The confirmed correct Bacmid-*wace*1 and Bacmid-*mace*1 plasmid DNA was transfected into *sf*9 cells using Celfectin transfection reagent (Life Technologies) following the manufacturer’s manual. After 3–4 days culture at 27 °C, correct growth and morphology of cells were confirmed under an inverted microscope. The *sf*9 cells transfected with wild type Bacmid and those without transfection were both used as negative controls. The supernatants of transfected cells were collected and saved at 4 °C, and the pellets were centrifuged at 300 g for 5 min to remove cells and debris to obtain the passage 1 (P1) virus. P1 virus was used to re-infect the cells to obtain high titer recombinant virus solutions. The virus titer and the 50 % tissue culture infective dose (TCID50 value, data not shown) were determined by using the end-point dilution method [23].

#### Detection of recombinant virus expression

The *sf9* cells with 72 h Bacmid infection (Bacmid-*wace*1 and Bacmid-*mace*1) were collected along with the negative control cells for SDS-PAGE. SDS-PAGE was performed using electrophoresis cells from Bio-Rad; the separating gel contained 12 % acrylamide, and the stacking gel contained 5 % acrylamide. Routinely, the gels were stained for proteins with Coomassie brilliant blue R250. After proteins were transferred from gels (120 min, 70 V) to PVDF membranes (Millipore, USA)by tank Blotting, the membranes were blocked for 1 h with a 5 % skim milk/TBST solution (0.02 M Tris, 0.15 M NaCl, 0.1 % Tween 20, pH 7.5) at 4 °C and then incubated with anti-His-tag antibody (diluted 1:5, 000) for 1 h at room temperature. After washing in TBST for 15 min for three times, the membranes were incubated for 1 h at room temperature with a goat anti rabbit IgG-AP (diluted 1:10, 000 in skim milk/TBST solution). After washing in TBST for 15 min for three times, the blots were stained with the AP Conjugate Substrate Kit following the kit protocol. Then the proteins were purified with the Ni–NTA purification system following the user manual.

#### Biological activity analysis

The activity assay was evaluated using the method of Ellman et al. [24] with some modifications. The reaction was carried out in 96-well microtiter plates. In detail, the activity of AChE was determined by the acetylcholinesterase measurement kit following the manufacturer’s manual (Nanjing Technology Co., Ltd.). The Substrate buffer was ATC(acetylthiocholine iodide) with different concentrations from 0.05 to 10 mM. Four groups of samples were used in the measurement procedure: the blank control group, the standard control group, the experimental control group and the experimental group. The volume of each component was following Table 2. All components were mixed and incubated for 10 min for measurements at 412 nm in 0.5 optical path. The activity of AChE = [(experimental group − experimental control) × AChE standard concentration]/[(standard control- blank control) × Experimental sample concentration]. Three biological replicates were used in each group. The signals were read with a Multiskan GO microplate reader (Model5111 9200; Thermo Scientific, Nanjing, China) at 412 nm, with one reading per 30 s. The reaction was at 30 °C, and 10 values were recorded for each reaction. The data were recorded and processed with the Skanit software (Thermo Scientific), and the values of Km and Vmax were calculated by using the Lineweaver–Burk plots method.

**Table 2:** The volume of each component in activity detection

	Experimental group	Experimental control	Standard control	Blank control
Experimental sample (μL)	10	–	–	–
AChE standard (μL)	–	–	10	–
Double distilled water (μL)	–	–	–	10
Substrate buffer (μL)	100	100	100	100
Color developing agent (μL)	100	100	100	100
Mixed and incubated at 37 °C for 6 min				
Inhibitor (μL)	6	6	6	6
Transparent agent (μL)	20	20	20	20
Stabilizer (μL)	10	10	10	10
Sample (μL)	–	10	–	–

In the detection of remaining activity, 10 μL enzyme solution was mixed with 10 μL physostigmine or phoxim solution at different concentrations in microtiter plates and settled at 37 °C for 10 min for complete binding of the enzyme and its inhibitor. Water was used as control. The remaining activity of AChE was then determined by using the acetylcholinesterase measurement kit following the manufacturer’s manual (Nanjing Technology Co., Ltd.). Three biological replicates were used in each group.

#### Statistical Analysis

All results are expressed as mean ± standard error (SE). The significant differences were examined by unpaired Student’s *t* test using SPSS 19 software (USA). A *p*-value <0.05 was considered as statistically significant.

## Results

### Directed mutagenesis

**Fig. 1:**
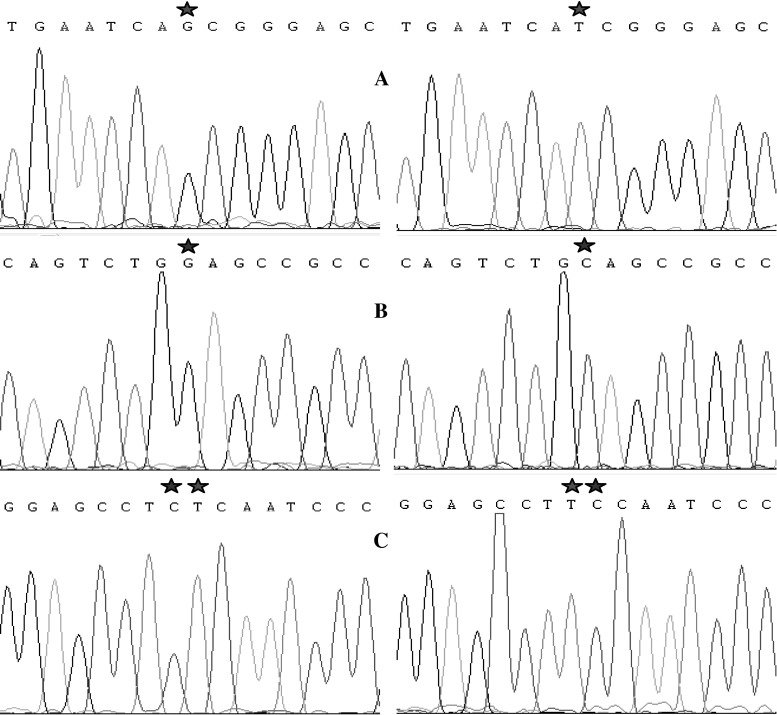
Sequencing figure of the *wace*1 and *mace*1 (*A*, *B*, *C* indicates the mutation site, and the asterisk indicates the mutation bases sites)

With plasmid pUC-*wace*1 as template, the mutant plasmid was generated by directed mutagenesis PCR, digested with *Dpn*I to eliminate the template, and transformed into *E. coli* DH5α competent cells. The positive colonies were picked and the plasmid DNA was amplified for sequencing. As shown in Fig. 1, the G at position 907 was mutated into T, the G at position 986 was mutated into C, and the C-T at positions 1, 660 and 1, 661 were mutated into T-C, which resulted in the amino acid changes of Ala303Ser, Gly329Ala and Leu554Ser.

### Construction and identification of pFastBac™ HT B-*wace*1 and pFastBac ™ HT B-*mace*1

**Fig. 2:**
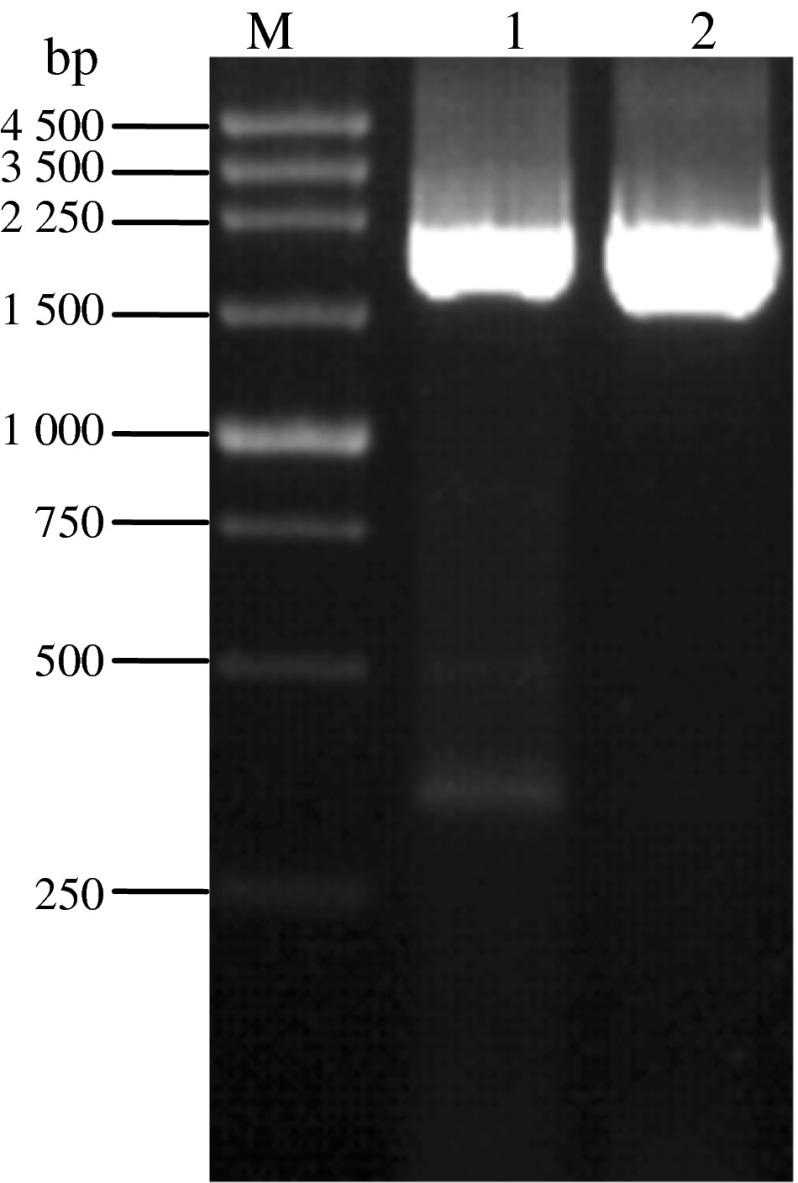
PCR results of wild type and mutant acetylcholinesterase genes M. 250 bp DNA marker *1* PCR product of *wace*1 *2* PCR product of *mace*1

**Fig. 3:**
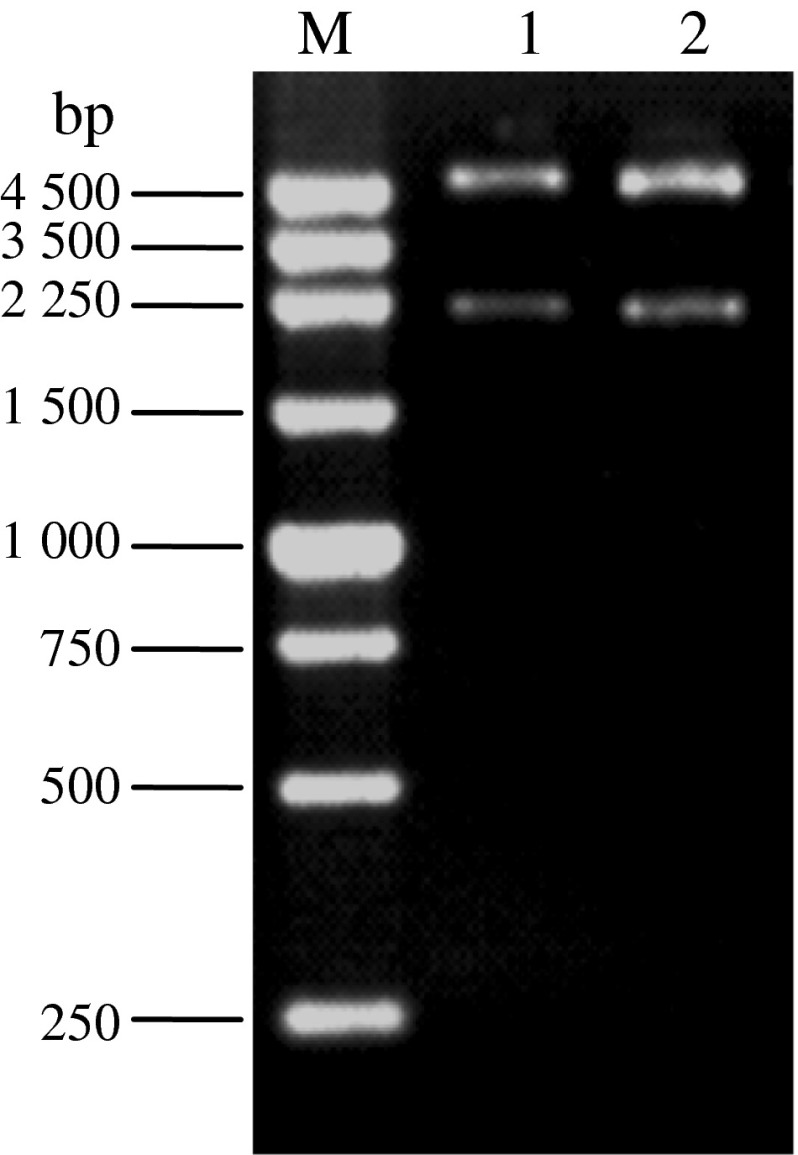
Verification of recombinant plasmid pFastBac™ HT B-*ace* 1 by enzyme digestion M. 250 bp DNA marker *1* pFastBac HT B-*mace*1 recombinant plasmid digested with *Xba* I and *Xho* I *2* pFastBac™ HT B-*wace*1 recombinant plasmid digested with *Xba* I and *Xho* I

The size of the *ace*1 gene is 2, 100 bp. As shown in Fig. 2, fragments with expected size of the *ace*1 gene from PCR amplification with pUC-*wace*1 and pUC-*mace*1 as templates were observed after *Xba*I and *Xho*I digestion. The size of the donor plasmid pFastBac™ HT B is 4.8 kb. As shown in Fig. 3, a band with expected size of about 2, 100 bp was observed from the *Xba*I and *Xho*I digestion of the recombinant plasmids pFastBac™ HT B-*wace*1 and pFastBac™ HT B-*mace*1, demonstrating the successful construction of pFastBac™ HT B-*ace*1.

### PCR identification of the recombinant baculovirus Bacmid-*ace*1

**Fig. 4:**
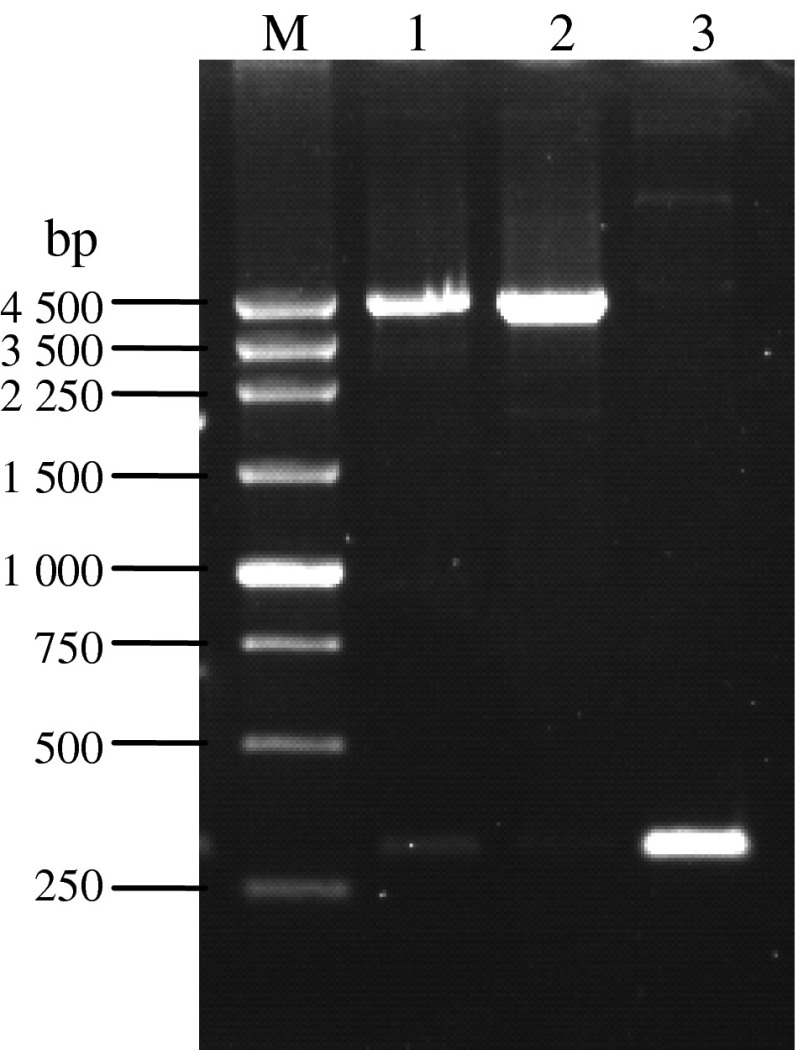
Verification of recombinant Bacmid-*ace*1 by PCR M. 250 bp DNA marker *1* PCR product of Bacmid- *wace*1 *2* PCR product of Bacmid- -*mace*1 *3* PCR product of the empty bacmid

Using the recombinant baculovirus plasmids Bacmid-*wace*1 and Bacmid-*mace*1 as templates, the targeting gene of *ace*1 with the size of 2, 100 bp was PCR amplified with the primer pair of M13 forward/M13 reverse; the successfully transposed Bacmid with the size of 4, 500 bp was PCR amplified with the primer pair of M13 forward/M13 reverse, while the PCR product for the Bacmid without transposition was only 300 bp. As shown in Fig. 4, expected sizes of PCR products were obtained, demonstrating the successful transposition of the *ace*1 gene.

### Transfection of recombinant baculovirus plasmids Bacmid-*wace*1 and Bacmid-*mace*1 into *sf9* cells

**Fig. 5:**
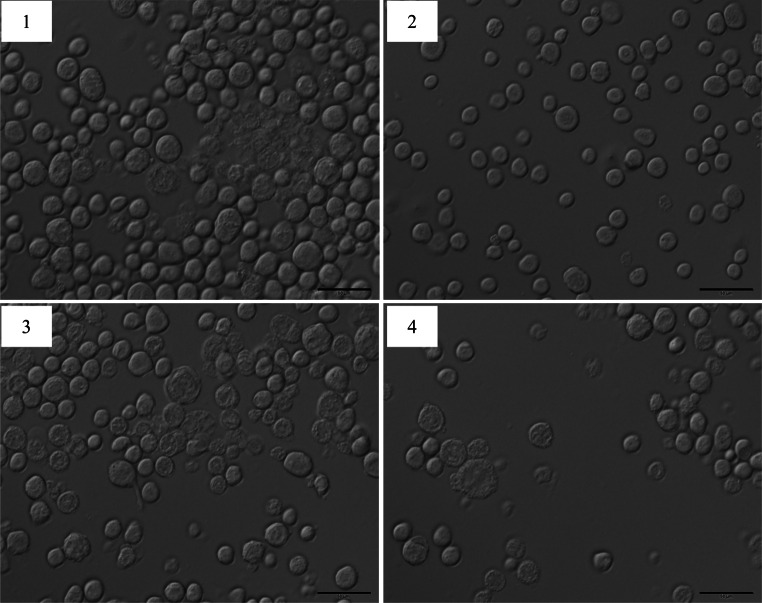
Phenotype of *sf*9 cells transfected with recombinant Bacmid-*wace1* and Bacmid-*mace1* under microscope (*scale bars* represent 50 μm). *1* Cells transfected with recombinant Bacmid-*wace*1; *2* sf9 cells; *3* cells transfected with recombinant Bacmid-*mace*1; *4* cells transfected with empty Bacmid. *sf*9 cells and cells transfected with empty Bacmid were used as controls

The identified recombinant baculovirus plasmids Bacmid-*wace*1 and Bacmid- *mace*1 were transfected into *sf*9 cells in logarithmic phase. 7 days after transfection, the cells were observed under an inverted microscope, which revealed significantly changes that included round shapes, enlarged nucleoli, the formation of intracellular vesicles and apoptotic bodies, and cell detachment and suspension (Fig. 5). The viral supernatants were collected and saved for re-inoculation. The *sf*9 cells were infected with the P3 virus, and the cells displayed apparent pathological symptoms 72 h later.

### Product detection of recombinant virus expression

**Fig. 6:**
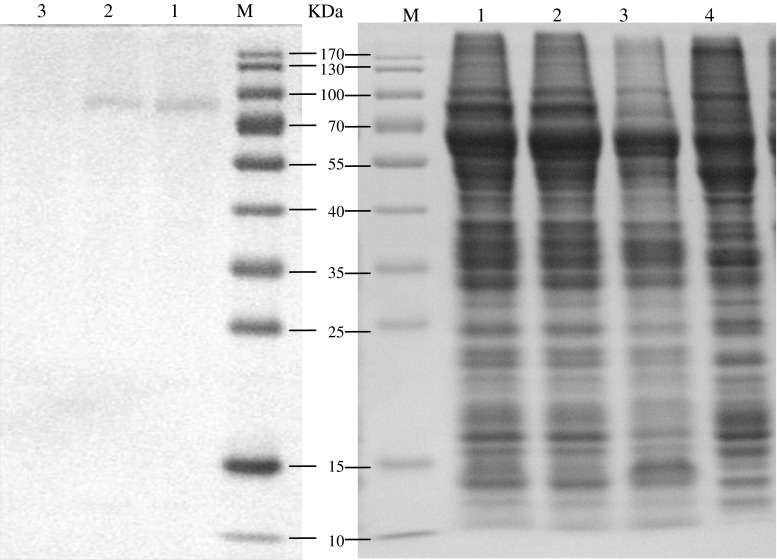
Analysis of recombinant ace1 expressed in *sf*9 cells : the *left picture* was the western blotting results of cells transfected with empty Bacmid, *mace*1 and *wace*1; the *right picture* was the SDS-PAGE of expression product (*M* prestained protein ladder, *1*–*4* *mace*1 expression product, *wace*1 expression product, control bacmid expression product, and *sf*9 cell expression product, respectively.)

**Fig. 7:**
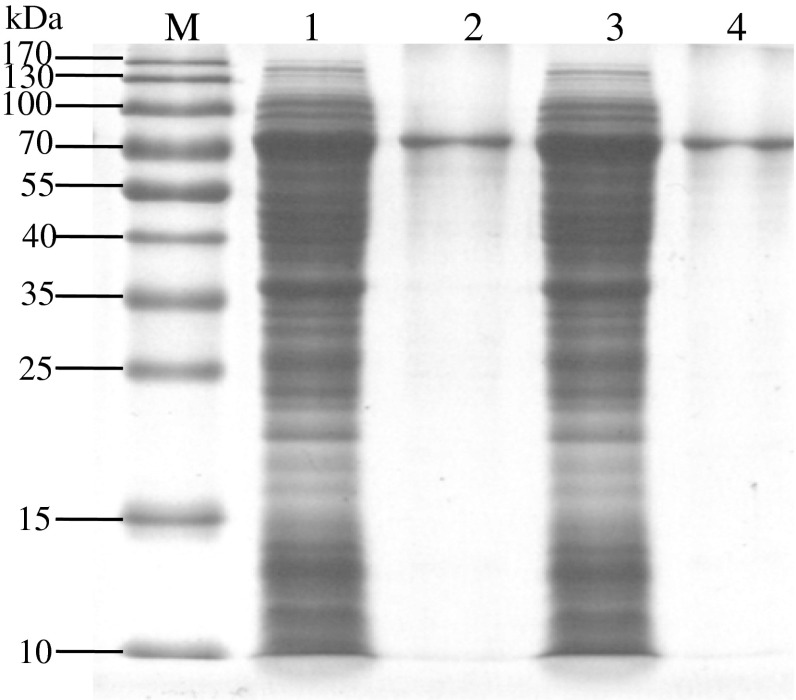
Expression of the *Bmace*1 in *sf*9 and purification *M* prestained protein ladder, *1* *mace*1 expression product, *2* purification product of *mace*1 expression product, *3* *wace*1 expression product, *4* purification product of *wace*1 expression product

*Sf9* cells were infected with the P3 recombinant viruses Bacmid-*wace*1 and Bacmid-*mace*1 (5 × 10^5^ cells/well) with empty virus as negative control. The cells were collected after 72 h culture for SDS-PAGE and Western-blotting analyses. As shown in Fig. 6, an expected specific band with the size of about 76 kDa was observed, demonstrating correct expression of the targeted protein. Approximately 0.53 mg of purified AChE was obtained from 100 mL culture solution, and SDS-PAGE analysis revealed only one band for the purified enzyme (Fig. 7), demonstrating sufficiently high purity of the purified AChE for further experiments.

### Biological activity of the expression product

**Fig. 8:**
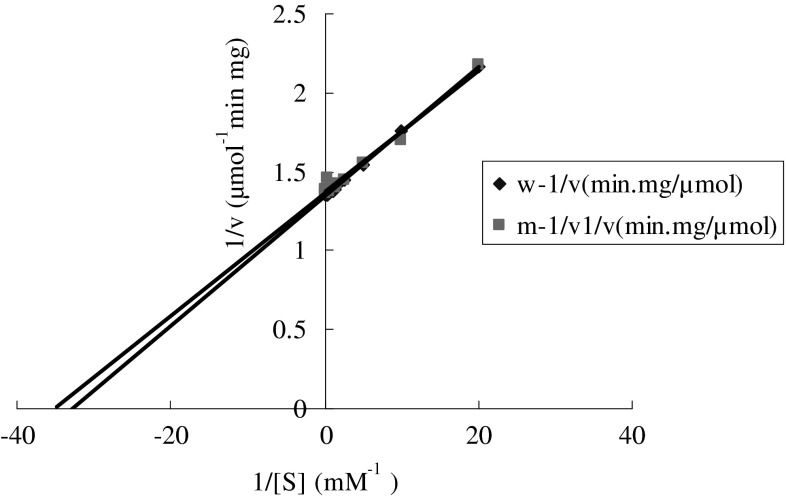
Lineweaver-Burk double-reciprocal plot of AChE activity for substrates acetylthiocholine (ATCh (X intercept = − 1/Km; Y intercept = 1/Vmax; Slope = Km/Vmax)

Recombinant AChE expressed in the baculovirus system was used for biochemical characterization. Biochemical kinetic assays (Fig. 8) revealed that the Km of wAChE and mAChE was 0.0305 mM and 0.0284 mM for acetylthiocholine, respectively, with the Vmax values of 0.731 and 0.743 μM min^−1^ mg^−1^, respectively, demonstrating the preference for acetylthiocholine substrates. These results indicate that the two enzymes have similar kinetic properties.

**Fig. 9:**
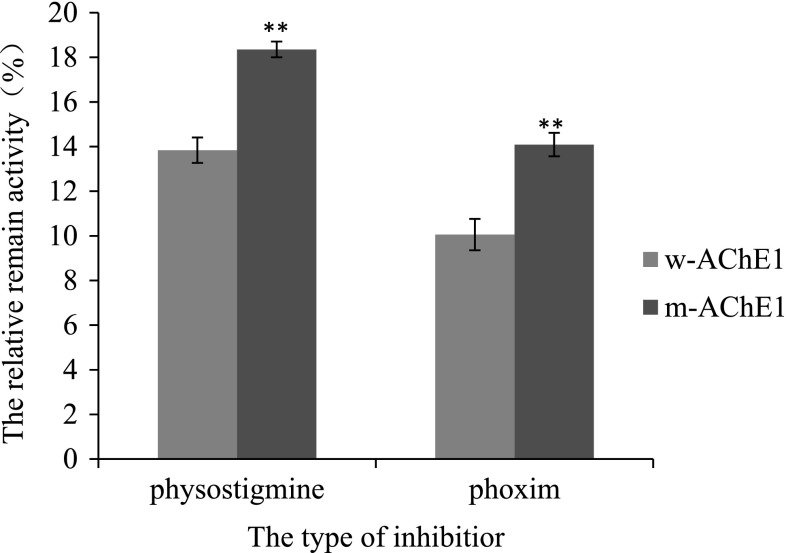
Inhibition plot for AChE activity with physostigmine and phoxim. (the physostigmine concentration was 10 μM; The phoxim concentration was 33.4 μM). *Bars* marked with *double asterisks* means it is significantly different from the w-AChE1. (*p* < 0.05)

The inhibition of the two AChEs by physostigmine and phoxim was investigated to reveal the effects of mutations on the enzymic activity. When the physostigmine concentration reached 10 μM, the relative remain activity of the wild-type AChE was 13.84 ± 0.56 %, only 75.41 % of that of the mutant AChE at 18.35 ± 0.71 %, indicating higher sensitivity of the wild type AChE to acetylcholinesterase inhibitors (Fig. 9). After being treated with 33.4 μM phoxim, the mutant AChE’s relative remain activity was 14.09 ± 0.53 %, about 1.40 times of the wild type AChE’s activity at only 10.06 ± 0.35 %, indicating that the wild type AChE is more sensitive to organophosphorus insecticides and that the mutant AChE has weaker binding ability to phoxim (Fig. 9).

## Discussion

### Relationship between *ace*1 function and mutations

Lepidopteran insects have two AChEs, the two enzymes have distinct catalytic properties and responses to different inhibitors, AChE1 is more sensitive than AChE2 to most OP insecticides[25].In this study, we generated mutations for three amino acids in AChE1, the site of Ala303 is located in the cholinesterase activity center (FGESAG) and close to the catalytic triad Ser302. Mutations were also discovered in similar region (Ala201Ser) in the resistant strains of diamondback moth [12], indicating that mutations in this site may affect the function of acetylcholinesterase. Other mutations, such as Gly303Ala and Gly262Ala, may be related to organophosphorus pesticide resistance in *Drosophila melanogaster* and *Musca domestica*, respectively [7, 26]. The Drosophila Gly303 that corresponds to the *B. mori* Gly329 has been reported to affect the orientation of Ser276, which is phosphorylated or arbamoylated by insecticides [7]. The Leu554 is located in the conserved region of AChE and is changed to Ser in the resistant strains of Diamondback moth. The findings in this study demonstrate the important roles of the three point mutations in the function of the *B. mori* AChE.

### Interaction among mutation sites

It has been reported that either Gly228Ser or Phe439Trp mutation in *Tetranychus urticae* increased the insensitivity of insects by 26 or 99 times, respectively, while double mutations were able to increase the insensitivity by 1 165 times. The Ala391Thr mutation alone did not change the dynamics, although it did inhibit the effects of Phe439Trp mutation [27], indicating an interaction between different *ace* mutations. In this study, the activity difference between the wild type and mutant AChEs was not significant, suggesting that the interactions among the three mutations may affect the enzymic activity. Further investigations are needed to explore the specific synergy or antagonism among the three mutations.

### Functional study of the *B. moriace*1 mutations

In recent years, the resistance of Lepidopteran insects to chemical pesticides has increased gradually with their widespread use in agriculture and forestry and has been the focus in the research of pest control. On the other hand, the *B. mori* is an important economic Lepidopteran insect with low resistance to chemical pesticides because of long-term indoor domestication. Therefore, chemical pesticide contamination has become a serious problem for the sericulture in China [28]. In this study, we constructed plasmids containing different mutant sites of the *ace* gene, based on the discoveries from the resistant Diamondback moth. The mutant AChE was expressed using the baculovirus expression system. The expressed protein was purified and inhibited by physostigmine and phoxim to measure the remaining enzyme activity. The mutant AChE showed significantly higher remaining enzyme activity than the wild-type, indicating the close relationship between the mutated sites and AChE’s sensitivity to physostigmine and phoxim. Due to the complexity of the metabolic resistance at organismic level, further investigations are clearly required to confirm the findings on the pesticide resistance in vivo.
